# Human bipedal instability in tree canopy environments is reduced by “light touch” fingertip support

**DOI:** 10.1038/s41598-017-01265-7

**Published:** 2017-04-25

**Authors:** L. Johannsen, S. R. L. Coward, G. R. Martin, A. M. Wing, A. van Casteren, W. I. Sellers, A. R. Ennos, R. H. Crompton, S. K. S. Thorpe

**Affiliations:** 1 Department for Sport and Health Sciences, Technische Universität München, Munich, Germany; 2 School of Health Sciences, University of East Anglia, Norwich, UK; 3 School of Biosciences, University of Birmingham, Birmingham, UK; 4 School of Psychology, University of Birmingham, Birmingham, UK; 5 Max Planck Weizmann Center for Integrative Archaeology and Anthropology, Max Planck Institute for Evolutionary Anthropology, Leipzig, Germany; 6 Faculty of Life Sciences, University of Manchester, Manchester, UK; 7 School of Biological, Biomedical and Environmental Sciences University of Hull, Hull, UK; 8 School of Biomedical Sciences, University of Liverpool, Liverpool, UK

## Abstract

Whether tree canopy habitats played a sustained role in the ecology of ancestral bipedal hominins is unresolved. Some argue that arboreal bipedalism was prohibitively risky for hominins whose increasingly modern anatomy prevented them from gripping branches with their feet. Balancing on two legs is indeed challenging for humans under optimal conditions let alone in forest canopy, which is physically and visually highly dynamic. Here we quantify the impact of forest canopy characteristics on postural stability in humans. Viewing a movie of swaying branches while standing on a branch-like bouncy springboard destabilised the participants as much as wearing a blindfold. However “light touch”, a sensorimotor strategy based on light fingertip support, significantly enhanced their balance and lowered their thigh muscle activity by up to 30%. This demonstrates how a light touch strategy could have been central to our ancestor’s ability to avoid falls and reduce the mechanical and metabolic cost of arboreal feeding and movement. Our results may also indicate that some adaptations in the hand that facilitated continued access to forest canopy may have complemented, rather than opposed, adaptations that facilitated precise manipulation and tool use.

## Introduction

Increasing recent evidence is challenging the long held concept that the evolution of bipedalism in early hominins was a key factor that resulted in a permanent shift from arboreal to terrestrial life. Instead ancestral bipedal hominins appear to have continued to exploit tree canopy habitats well into our own genus *Homo* (e.g. refs 1–4). However, human bipedal stance is an inherently unstable posture^5^ and the forest canopy is highly dynamic, which presents serious challenges for bipedal balance and movement. Together these raise fundamental questions about how bipedal hominins managed to exploit arboreal habitats with increasingly modern morphologies, which are central to understanding the environmental influences that have shaped modern human anatomy.

In humans the Centre of Mass (CoM) of the body lies further forward than the ankles so that, even in quiet standing on a stable support, the muscles of the calf must exert a torque to stop the body toppling forwards^5^. This torque may need to be significantly increased when balance is disturbed. Moreover, the central nervous system perceives self-motion and motion of the environment simultaneously via sensory cues from vision, the vestibular system, proprioception in the leg muscles, and tactile information from the soles of the feet^6^. Deprivation of one of these sensory cues or conflicting messages between them causes conspicuous instability and body sway^7, 8^. Thus bipedalism for early hominins exploiting tree canopy habitats would have been particularly challenging since branches typically flex under the body mass of large animals, which destabilises the body^9, 10^. The visual environment of forest canopy is also dynamic and unpredictable as branches move in the wind and under the weight of other animals. This feature of forest canopy has not been considered previously as influential on arboreal balance in humans or other primates. However, it has been established that irregularly-moving virtual visual environments are particularly challenging to human balance, and cause the central nervous system to initiate inappropriate muscle activation patterns while it distinguishes between movement of the body and movement of the environment^11, 12^.

Non-human great apes can circumvent the problems of arboreal balance by hanging from their long hands or by using arboreal bipedalism, stabilised by their long prehensile toes that can grip to oppose the toppling moment experienced when standing on a thin branch^9, 13^. In contrast many early hominins had short hands that were unsuitable for prolonged suspension^14–17^ and while the foot of *Ardipithecus ramidus* and the Burtele foot (BRT-VP-2/73, Woranso-Mille, Ethopia) indicate that these species had some residual gripping ability^18, 19^, the foot morphology of *Australopithecus afarensis* and the Laotoli footprints suggests that from 3.66 million years ago (MYA) many hominins were exploiting the forest canopy with essentially modern human-like feet that were not capable of gripping branches^20, 21^.

The aim of this study was to investigate how modern humans achieve bipedal stability in a controlled environment that embodies both the physical and visual challenges of forest canopy environments. We propose that a sensorimotor mechanism based upon “light touch” could have enabled early bipedal hominins to counter the physical and visual challenges of forest canopy.

Light touch refers to sensory feedback received from the surface of the fingers^22^. It has been suggested that bipedalism could be mechanically more stable in the forest canopy than on the ground because it would be possible for bipeds to use their hands to hold branches to aid balance^23^. But the flexibility of most available hand supports in forest canopy is instead likely to destabilise the body if significant loads are placed upon them. In contrast, touching a surface very lightly with a finger, similar to the touch used in Braille letter recognition^24^ provides a sensorimotor feedback strategy that can enhance human stability without the large forces required for mechanical support. This light touch effect is produced by cutaneous receptors sensing small differences in shear forces through skin deformation^5, 25^ and it substantially increases postural stability on firm supports during quiet standing and after a perturbation to balance^8, 25–27^. It has also been shown to improve gait efficiency by reducing muscle activity in the lower leg during treadmill walking before and after a gait perturbation^28^. However, no study has assessed if it is beneficial in environments where participants are exposed simultaneously to challenging physical and visual environments, and where the structures available for the provision of tactile information are highly flexible.

**Figure 1:**
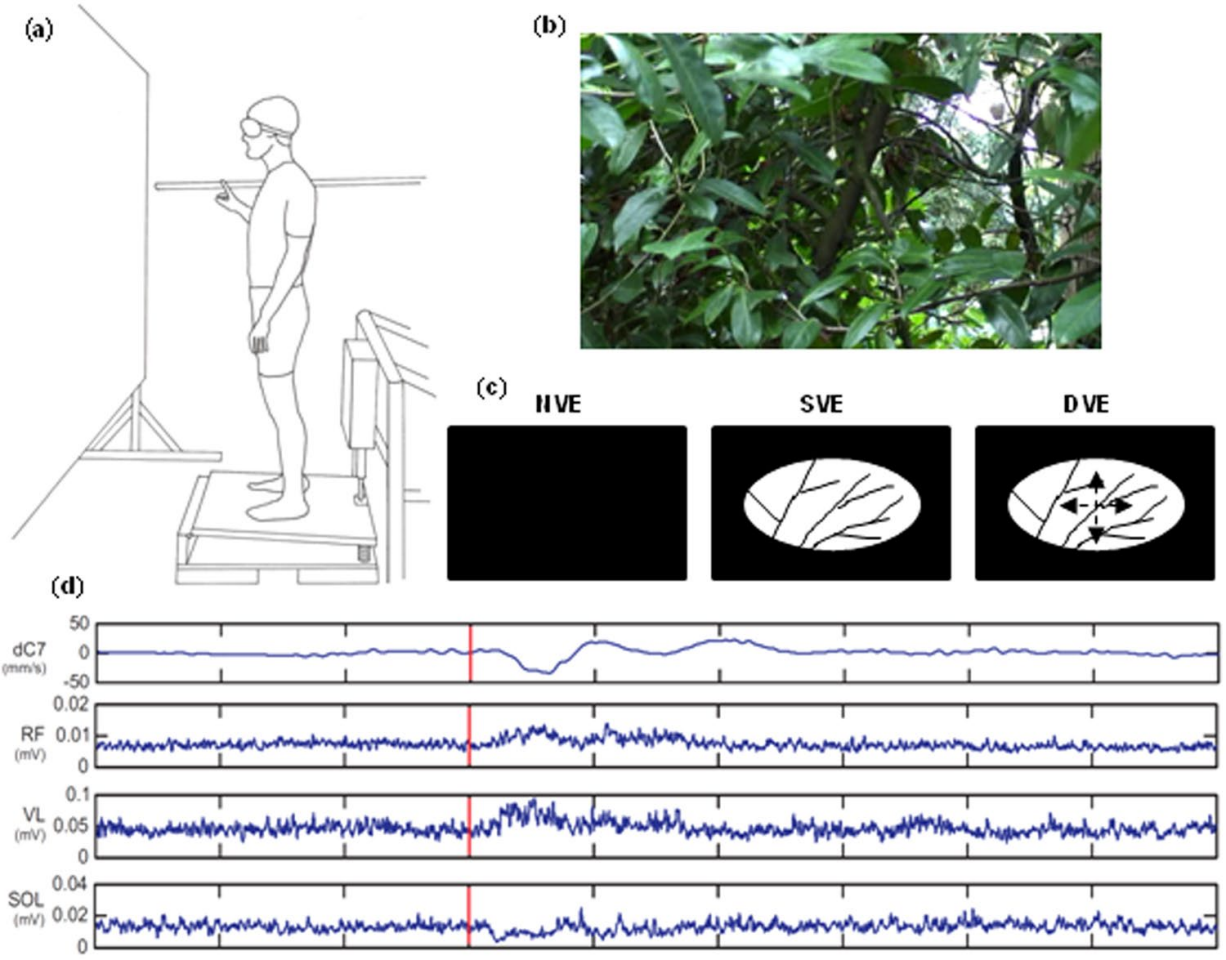
The experimental setup. (**a**) a participant on the springboard. The right arm is in a raised posture in contact with the compliant hand support. An actuator system is tracking the motion of the springboard ready to deliver a vertical perturbation. The participant wears goggles restricting their field of view to a back projection screen. (**b**) A still frame from the video used for the experiment. (**c**) The three visual environment conditions: NVE: no visual environment (participants wore a blindfold); SVE: static visual environment (a still frame from the video of the branches of a leafy tree); DVE: dynamic visual environment (the video of the branches). (**d**) Participant-averaged sample data traces for the velocity of body sway (dC7) and EMG from the rectus femoris (RF), vastus lateralis (VL) and soleus (SOL) muscles from 3 s before to 6 s after the perturbation. The solid red vertical line indicates the time point of the perturbation.

To investigate human stabilisation mechanisms in arboreal-like habitats we studied body sway and leg muscle activity of human participants standing barefoot on a cantilevered springboard. We first compared body sway responses and leg muscle activation levels of humans in quiet standing on the springboard when it was firm (where a solid chock replaced the springs) and when it was compliant. For compliant trials we then applied a mechanical vertical perturbation to the springboard to destabilise the participants. Secondly, we tested the effects of light touch on the participant’s postural stability and muscle activity levels during quiet standing and stabilisation after the mechanical perturbation. For all conditions we further exposed participants to different visual environments using a visual display system onto which images were back-projected (see Fig. 1). The visual environments were a static visual environment (SVE) consisting of a single frame of a video of the branches of a leafy tree, a dynamic visual environment (DVE) in which they viewed the video of the swaying branches, and wearing a blindfold in which there was no visual environment (NVE).

## Results

### Impact of dynamic physical and visual environments on quiet standing

**Figure 2:**
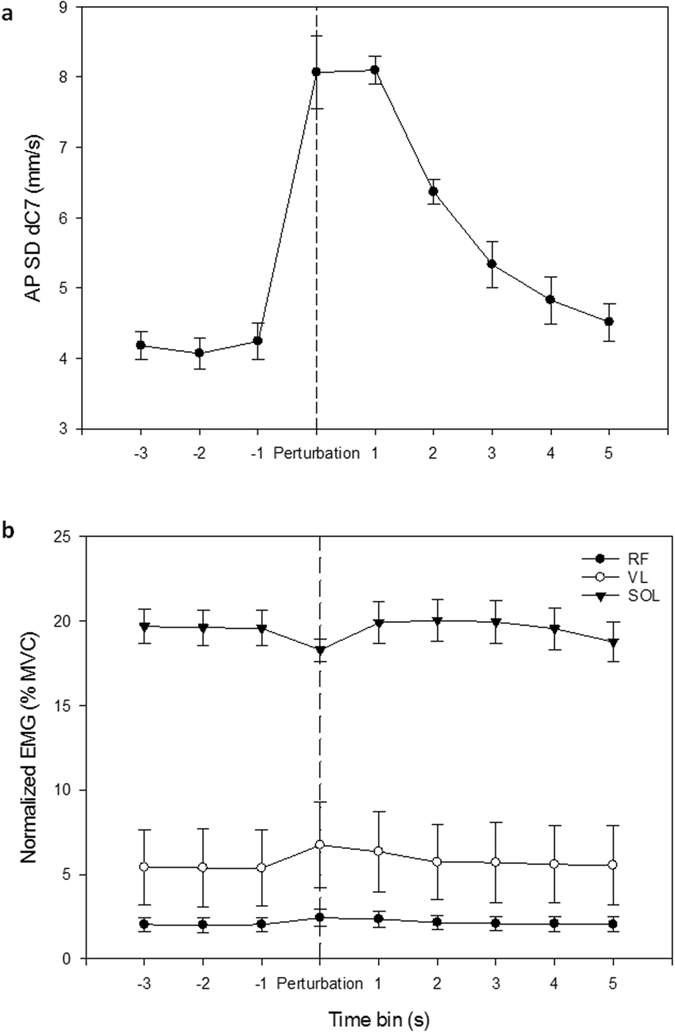
Time series for all participants across all support and visual conditions for measured variables. (**a**) the variability of antero-posterior velocity of body sway (SD dC7) and (**b**) average muscle activity (normalised to the participant’s maximum voluntary contraction, MVC). The muscles are rectus femoris (RF), vastus lateralis (VL) and soleus (SOL). Time equates to 1 s time bins from 3 s before to 6 s after the perturbation (see methods for description of time bins). Error bars represent standard errors.

**Figure 3:**
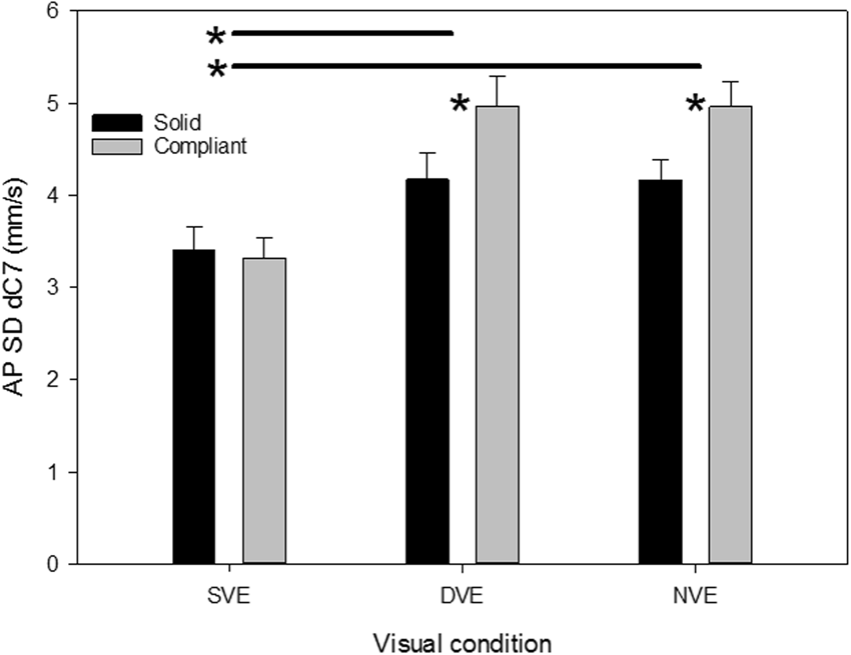
The effect of different visual environments and substrate compliance on antero-posterior (AP) body sway during quiet standing before the mechanical perturbation. Body sway is presented as the standard deviation (SD) of the variability of body sway. SVE: static visual environment; DVE: dynamic visual environment; NVE: No visual environment. *P < 0.05. Horizontal black lines with asterisks indicate significant post-hoc comparisons between visual conditions depicted on the horizontal axis, while single asterisks indicate significant post-hoc comparisons between both surface conditions within a specific visual condition.

The effect of viewing the dynamic visual environment was to destabilise the body as much as having no visual information available (Table 1; Fig. 2a and Fig. 3). When standing on the solid support the variability of antero-posterior body sway velocity measured at the 7^th^ cervicular vertebrae in the neck (SD dC7, hereafter ‘body sway’), which is a strong indicator of the postural control system’s “effort” to stabilize balance^29^, was 22% greater for both DVE and NVE compared to the SVE (Fig. 3). Moreover, the interaction between challenging visual and support conditions further decreased stability since for NVE and DVE conditions body sway was significantly increased when standing on the compliant support compared to the solid support (20% for both conditions), whereas participants were equally stable on both surfaces when they viewed the SVE (Table 1; Fig. 3). Activity levels in all tested muscles were unaffected by visual and support conditions in quiet standing.

**Table 1:** Results from a GLM comparison of muscle activation and postural sway measures for quiet standing conditions in the antero-posterior direction.

Position/Measure	P value					
	Touch F(1,6)	Vision F(2,12)	Compliance F(1,6)	Touch x Vision F(2,12)	Touch x Compliance F(1,6)	Vision x Compliance F(2,12)
Body sway	<0.001	<0.001	0.03	NS	NS	0.02
EMG_RF_	0.02	NS	NS	NS	NS	NS
EMG_VL_	NS	NS	NS	NS	NS	NS
EMG_SOL_	0.04	NS	NS	NS	NS	NS

Body sway was measured at the level of the base of the neck (7^th^ cervicular vertebrae). NS: no significant difference. Three-way interactions were not studied due to the sample size.

### Impact of dynamic visual environments after a support perturbation

**Figure 4:**
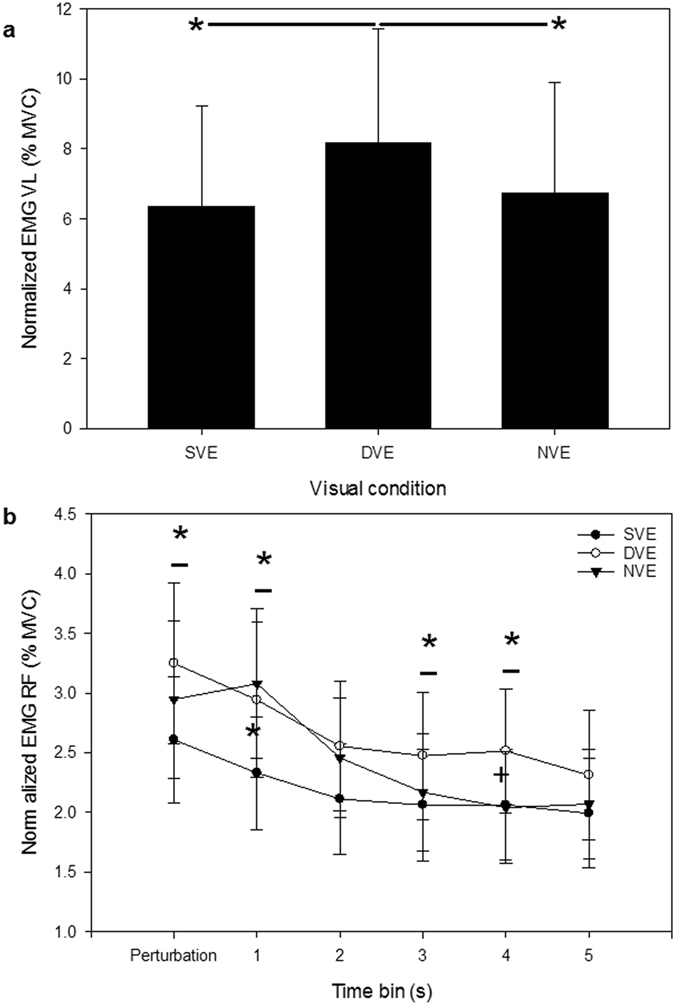
Influences on muscle activity after the perturbation. (**a**) The effect of different visual environments on vastus lateralis muscle activity after the mechanical perturbation. (**b**) The effect of visual environments and time on rectus femoris activity levels after the mechanical perturbation. Muscle activity is presented as the percent of the participants’ maximum voluntary contractions (MVC). Horizontal black lines with asterisks indicate significant post-hoc comparisons between visual conditions. SVE: static visual environment; DVE: dynamic visual environment; NVE: No visual environment. See methods for description of time bins. *P < 0.05.

The participants body sway increased by 400% when they experienced the mechanical perturbation (Fig. 2a). Body sway began to stabilise after 2 seconds (i.e. in the 2nd time bin after the perturbation), but had not returned to the level of quiet standing after 6 seconds (Table 2 and Fig. 2a). The perturbation also affected muscle activation levels in the vastus lateralis (Fig. 4a) and the rectus femoris (the latter via an interaction with time) (Fig. 4b). Activity in the vastus lateralis was significantly higher for the stabilisation phase when viewing the DVE than when viewing the SVE (29%) or when participants were blindfold (22%) (Fig. 4a). Peak levels of rectus femoris muscle activity occurred within the perturbation time bin for the SVE and DVE, but one to two seconds after the perturbation (time bin 1) when no visual information was available (Fig. 4b). Thereafter, rectus femoris activity was lowest for the SVE trials. It reduced rapidly in the NVE trials to the same final level as for the SVE, but was significantly higher for the DVE immediately following the perturbation (15%) and in the 3^rd^ and 4^th^ time bins after it (26% and 45% respectively) (Fig. 4b).

**Table 2:** GLM comparison of muscle activation and postural sway in the antero-posterior direction for post perturbation conditions.

Measure	P value					
	Touch F(1,6)	Vision F(2,12)	Time F(5,30)	Touch x Vision F(2,12)	Touch x Time F(5,30)	Vision x Time F(10,60)
Body sway	0.001	<0.001	0.001	0.002	NS	NS
EMG_RF_	0.03	NS	0.01	NS	NS	0.04
EMG_VL_	0.03	0.009	0.01	NS	0.04	NS
EMG_SOL_	NS	NS	0.03	NS	NS	NS

Body sway was measured at the level of the base of the neck (7^th^ cervicular vertebrae). NS: no significant difference. Three-way interactions were not studied due to the sample size.

### The effect of light touch

**Figure 5:**
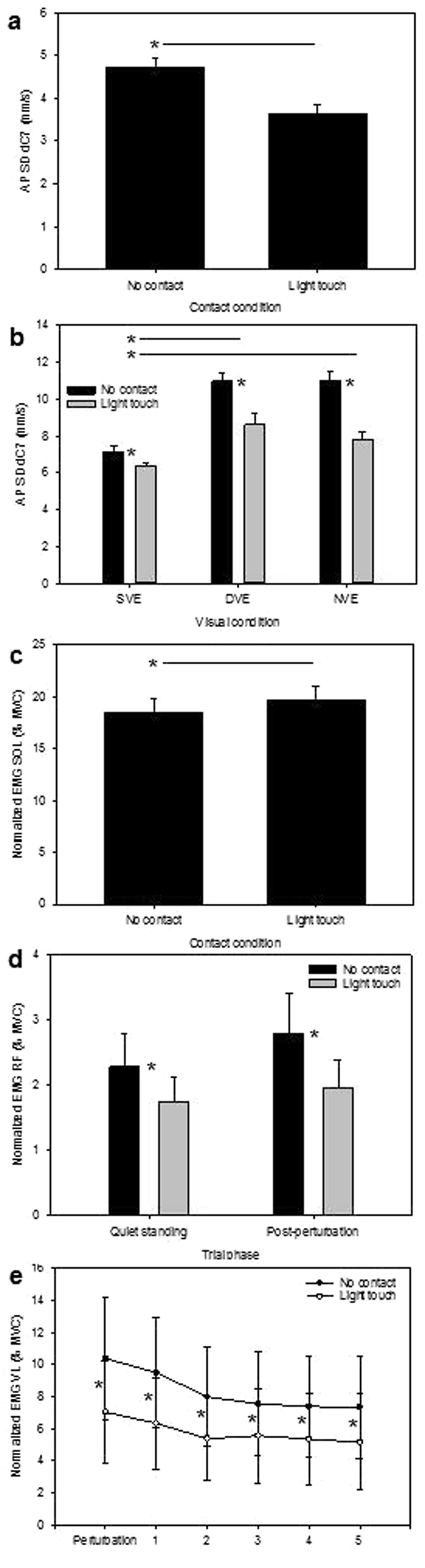
The effect of light touch on: (**a**) body sway in quiet standing, (**b**) body sway after the perturbation according to visual condition, (**c**) soleus activity in quiet standing, (**d**) rectus femoris activity in quiet standing and after the perturbation and e) vastus lateralis activity after the perturbation, according to time. MVC: maximum voluntary contractions. SVE: static visual environment; DVE: dynamic visual environment; NVE: No visual environment. See methods for description of time bins. *P < 0.05. Horizontal black lines with asterisks indicate significant post-hoc comparisons between major conditions depicted on the horizontal axis, while single asterisks indicate significant post-hoc comparisons between subconditions within a specific major condition.

The average resultant light touch force for the hand was 0.29 N (SD: 0.31 N) during the quiet standing phase and 0.33 N (SD: 0.33 N) during the stabilization phase of the trials. Lightly touching the compliant hand support reduced body sway in quiet standing by 24% compared to when participants had to balance without touch (Table 1, Fig. 5a). After the perturbation on the compliant support body sway was affected by the interaction between light touch and vision (Table 2): light touch significantly reduced sway for all visual conditions, however its effect for the DVE and NVE was substantially larger (22% and 29% respectively) than for the SVE (11%), while its impact in DVE and NVE did not differ significantly from each other (Fig. 5b). Light touch had no effect on the time it took the participants to stabilise (Table 2).

Light touch also affected muscle activity levels. Soleus muscle activity was significantly elevated (6%) in light touch compared to no touch trials in quiet standing (Table 1, Fig. 5c), but there was no effect after the perturbation (Table 2). This effect was reversed for the thigh muscles. Light touch significantly reduced rectus femoris activity levels in quiet standing (Table 1) and both rectus femoris and vastus lateralis activity levels (the latter via an interaction with time) after the perturbation (Table 2), compared to no touch trials. Rectus femoris activity was 23% less in light touch trails than in no touch trials during quiet standing (Fig. 5d). After the perturbation it was 32% less active with light touch than without (Fig. 5d). Vastus lateralis ranged from 30–32% less active in light touch than no touch trials after the perturbation (Table 2, Fig. 5e).

## Discussion

We present novel empirical data that quantifies the impact of the physical and visual challenges characteristic of forest canopy on postural stability in modern humans. We show that light touch makes a substantial difference to human’s basic ability to balance in forest canopy environments. Such ability could have underlain our ancestor’s success in arboreal locomotor, foraging and predator avoidance behaviours.

The results firstly confirm that, like virtual abstract visual environments^11, 12^, the visual environment of forest canopy does significantly destabilise humans. The impact on postural stability of the dynamic forest environment combined with standing on the compliant support was as severe as when the participants wore a blindfold. When the visual, vestibular and somatosensory senses provide unreliable and potentially conflicting information, central mechanisms can employ multi-sensory re-weighting to prioritise the input that offers the most reliable source of sensory information about own body sway^30^. The similarity in the sway response for being blindfold and viewing the dynamic visual environment suggests that vision was ‘downweighted’ in the latter to reduce its destabilising impact, amounting to a de facto deprivation of visual feedback. For forest canopy conditions however, this clearly creates a problem because vestibular and proprioceptive information on body sway will also be compromised by the compliance of available weight bearing branches; indeed we found that body sway was significantly higher for both DVE and NVE on the compliant support compared to the stiff support (Fig. 3). It also had a substantial effect on thigh muscle activity since rectus femoris was 40% more active (averaged over all time bins) and vastus lateralis 29% more active in DVE than SVE after the perturbation, and 17% (RF) and 22% (VL) more active in the DVE than the blindfold condition (Fig. 4a and b). Thus, the central nervous system in this experiment reacted in a similar way to its response in virtual visual environments^11, 12^; by initiating inappropriate muscle activation patterns while it distinguished between movement of the body and movement of the naturalistic environment.

**Figure 6:**
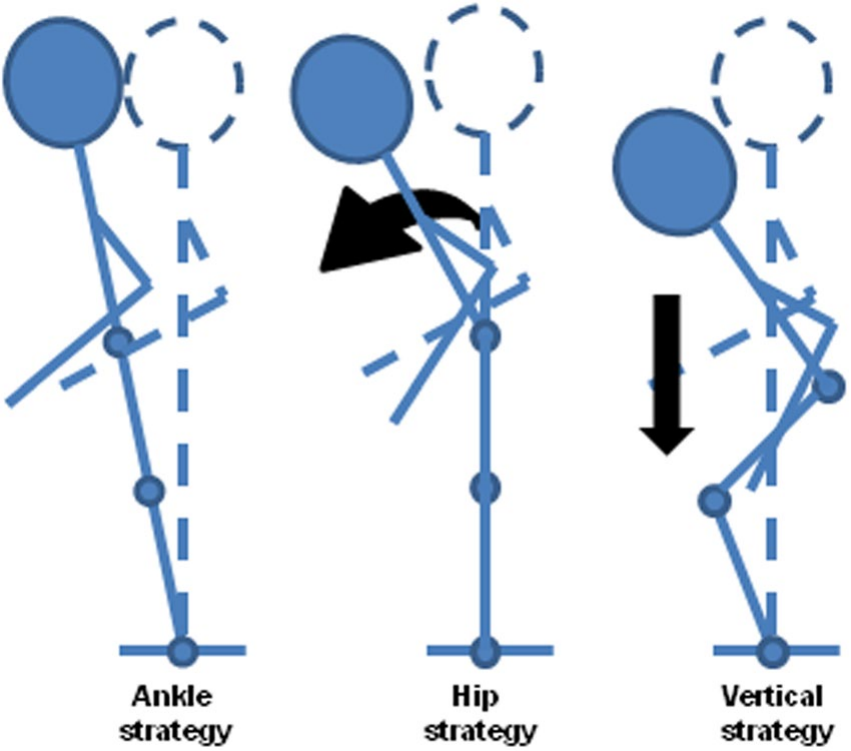
Postural response strategies for maintaining balance following a perturbation (modified after^35^). In the ankle strategy the toppling moment on the body is countered by a torque around the ankle joint, with the upper body acting as a single inverted pendulum. In the hip strategy a double inverted pendulum is created by an additional torque at the hip. In the vertical strategy all lower limb joints flex to control vertical height to counter the toppling moment.

Previous studies have shown that postural response strategies for maintaining balance following an external perturbation differ according to the environmental context, such as the nature of the threat to postural stability, the available sensory feedback and the specific features of the support^31^. The ‘ankle strategy’ is the most common strategy for controlling body sway in the anterior-posterior direction^32^ particularly on large, flat supports. In this strategy the toppling moment is countered mainly by the production of a torque around the ankle joint, with the upper body behaving as a single inverted pendulum^33^. In the forest canopy this might be encountered when standing along a single large but flexible branch; along two narrower branches (one per foot) or on ‘webs’ of intermingled narrow branches that form small, flexible platforms (all these are commonly used by wild orangutans^13^). When the postural context becomes more challenging a hip strategy may be also recruited, which creates a double inverted pendulum allowing for additional leaning of the upper body to stabilise body sway^34^, or a vertical strategy where the hip, knee and ankle flex to control vertical height^35^ (Fig. 6). In forest canopy these are likely to be elicited by standing astride one or more narrow branches. In this study, the soleus was the most active muscle in all trials (Fig. 2b), which suggests that the ankle was important in maintaining postural equilibrium throughout the experiment. However, the increased activity levels for RF and VL immediately after the perturbation (Fig. 4), and particularly in the DVE compared to both SVE and NVE show that forest canopy environments are sufficiently challenging to warrant more complex hip and vertical stabilisation mechanisms, which probably relate to an immediate response strategy to dampen the vertical oscillations of the branch after it has been disturbed.

We subsequently tested whether light touch on a compliant hand support might provide sufficient additional proprioceptive and cutaneous feedback from the fingers to reduce the destabilising effect of the dynamic physical and visual environment without destabilising the body by displacing the hand support. Even though we made the hand support in this study highly flexible (with an effective stiffness of only 1.17 N/mm at the participant’s point of contact with the pole), we found that light touch reduced body sway by 24% in quiet standing, independent of the visual and support conditions (Fig. 3). After the perturbation it significantly reduced sway for all visual conditions, but particularly for the dynamic visual environment and when blindfolded (22% and 29% respectively, Fig. 5b). In studies in which participants are able to lightly touch a solid hand support, it is commonly found that light touch decreases postural sway to the level found for the non-challenging condition, such as in dark compared to lit conditions^36, 37^. In this study, light touch countered just under two thirds (61%) of the combined destabilising effect of the dynamic visual environment and compliant support on postural sway (measured as the difference between the dynamic and static visual environment for no touch trials after the perturbation, Fig. 5b). This presumably reflects the complexity of forest canopy that creates multiple, simultaneous challenges for the sensorimotor system.

It might be thought that the participants could have been balancing with light mechanical touch rather than sensory support from the fingertips^38^. A rough calculation clearly shows this was not the case. The 7^th^ cervical vertebra moved with a peak velocity of 0.03 m sec^−1^ in the second immediately after the perturbation. This created a maximum possible displacement within that second of 16 mm at the height of the CoM, and hence caused a maximum toppling moment of about 12 Nm (calculated as displacement in m sec x gravity x participant’s body mass). In contrast, the hand forces, also exerted within 1 s after the perturbation, averaged around 0.3 N. The fingertip touched the hand support at shoulder height (1.5 m), and therefore acted at a large mechanical advantage. Nevertheless it would only have generated a mean restoring moment of about 0.5 Nm; just 4% of the toppling moment caused by the participants body sway. Thus the sway reductions seen with light touch cannot be explained by the amount of mechanical support and must have been caused largely by augmented sensory feedback giving faster feedback that the person was overbalancing, so allowing the ankle and hip muscles to respond quicker and reduce the forces they need to apply.

The relative impact of light touch was most powerful for activity in the upper leg where it reduced rectus femoris and vastus lateralis muscle activity by >30% after the perturbation(Fig. 5d and 5e). Activity levels in these muscles were lower overall than the soleus. However, our use of a flat surface rather than a branch structure was a simplified experimental paradigm that enabled us to compare relative underlying stabilisation mechanisms in different environmental conditions by ensuring that participants did not fall in the most challenging trials. It will nevertheless likely underestimate the instability and muscle activation levels that would be generated around the hip when standing on curved branch-like structures, which are likely to generate greater hip contribution and vertical strategies to control the stability of body balance. Thus if these muscles are representative of the quadriceps as a whole, and particularly if the light touch effect extends to locomotion, then light touch could be central not just to enhancing balance and avoiding falls in forest canopy habitats, but to significantly reducing the mechanical and metabolic cost of arboreal bipedality.

No study has directly quantified whether non-human great apes employ light touch to balance in the forest canopy. Orangutans exhibit higher levels of arboreal bipedality than the other great apes but the forelimbs appear to support more than their own weight in the majority of bipedal bouts^13, 23^. Chimpanzees also rely strongly on gripping feet and hand assistance^39^ during arboreal bipedality, suggesting neither species regularly utilises light touch in this behaviour. This may be because the long length of their arms, hands and fingers (particularly in orangutans) means that they can reach further to find suitable supports for the hands. They can also grip multiple small branches at once, which should provide a stiffer hand contact than the single flexible branches likely available to short-handed bipeds. Gorillas however are more similar to hominins in their short finger proportions (when scaled to body mass)^40^ and in their foot morphology^41^. Moreover they have the largest body mass of all great apes, which will increase the compliance of the branches used to bear their weight. This indicates they may experience somewhat similar balance challenges as hominins in forest canopy, and may therefore also employ light touch during arboreal bipedalism. Unfortunately of all the modern great apes, their arboreal locomotion is the least well documented.

The extent to which the performance of ancestral hominins in the forest canopy was compromised by climbing and clambering with modern foot morphology is currently unknown^42^. There is, however, strong evidence that *Au. afarensis* exploited both terrestrial and arboreal habitats^43^, despite possessing transverse and medial arches in the foot^21^ and modern human foot function, albeit less strongly expressed than in ourselves^20^. Other australopithecines such as *Au. africanus* (3–2 MYA) and *Au. sediba* (1.98 MYA) have also been shown to be competent terrestrial bipeds that retained a significant degree of arboreality^2, 3^. Within *Homo*, *Homo naledi* (date unknown) combines adaptations of the shoulders and hands that appear well suited for climbing with human-like features of the feet and lower limbs^44–46^, while cross sectional bone strength measurements on the humerus and femur indicate that *Homo habilis* (*circa* 1.8 MYA) also combined terrestrial bipedalism with frequent arboreal behaviour^47^. Nevertheless, ancestral hominins that retained short hands^40^ whilst undergoing adaptation of the feet for terrestrial bipedalism would need to evolve mechanisms to counter the instability caused by both the physical and visual dynamics of forest canopy if they were to maintain exploitation of forest resources without grasping feet. We suggest light touch, as a sensorimotor strategy, could have substantially enhanced balance stability without pedal grip to have improved safety, decreased the risk of falls, and decreased (or at least prevented large increases in) the mechanical and metabolic cost of arboreal locomotion.

In this scenario light touch would enhance balance during horizontal locomotion along and between flexible branches in the tree canopy, during foraging and in other arboreal behaviours. Nevertheless, hominins would still have needed to transition between the forest canopy and the ground using vertical climbing and descent. The curved phalanges, that are present in at least some fossil hominins (such as *Au. afarensis, Au. sediba* and *H. naledi* ^14, 45^ *)*, may well have enhanced efficacy and safety in this behaviour.

Our results may also have implications for the evolution of hominin hand morphology and sensorimotor functions of the central nervous system. Although all apes are capable of making contact between the tip of their thumb and their fingers, and thus forming precision grips, the ability to form pad to pad precision grips in which objects are held delicately yet securely between the proximal pulp surfaces of the thumb and the finger tips is present only in humans^48–50^. It has commonly been asserted that the precise manipulative hand morphology required for lithic tool use could have only been attainable after the hands had been freed from the constraints of arboreal locomotion. However, there is increasing evidence that early hominins, such as *Au. africanus* and *Au. afarensis*, were capable of forceful pad to pad precision grasping, even prior to the appearance of stone tools in the archaeological record^15, 51^. We suggest that hominin fingers might have been under selective pressure for light touch as an aid to balance in parallel with selection for the ability to perform forceful precision grips. Indeed, it is highly likely that fingers capable of using light touch are linked functionally to fingers capable of generating the high precision forces required for tool use because both rely on mechanoreceptive afferent fibers in the glabrous skin of the hand for tactile acuity. These fibers include fast adapting types associated with Meissner corpuscules and Pacini receptors, and slow adapting types associated with Merkel receptors^52^. Meissner corpuscles are thought to be particularly associated with tool use because they provide important sensory feedback for the effective control of grip and are especially numerous in the fingertips^53, 54^. Both fast adapting receptor types and Merkel cells have been shown to be integral to light touch, because their small receptive fields enable transmission of spatial details with a relatively high resolution^52, 55, 56^.

Comparisons between primates so far have, however, not revealed differences in the size or density of Meissner Corpuscules that would reflect human’s unique precision grip abilities^53, 54^. This may be due to methodological issues, for example, the use of an elderly human sample may have distorted the results since tactile acuity, particularly at the fingertip, deteriorates with age^52^. In addition Merkel cells were not studied, but these are also numerous in the fingertips and exhibit greater sensitivity than fast adapting receptors to non-uniform spatial features on objects (gaps, edges and curvatures)^57^. They are therefore considered to be critical for form and texture perception at the fingertip^52, 57^. However, a comparison of younger and older adult humans also showed that while reduced tactile sensitivity correlated with increased contact forces during light touch stance, the sway reduction by light touch itself did not vary with the contact force^52, 56^. Together these observations indicate that tactile sensitivity alone does not predict ability for the utilization of light touch for balance or for precision grip. Such features must be viewed in parallel with the higher order cognitive functions that process motor and tactile information of the hands (e.g. see ref. 58), integrate this information with other sensory cues, and/or resolve conflicting sensory messages in a specific postural context.

In summary, our data allow a unique insight into the sensory ecology of ancestral bipedal hominins. They add weight to the argument that exploitation of arboreal resources situated on peripheral, flexible branches would have been possible for hominins, despite their increasingly modern foot morphology. They may also indicate that some adaptations in the hand that facilitated continued access to forest canopy habitats may have complemented, rather than opposed, adaptations that facilitate precise manipulation and tool use.

## Methods

### Participants and apparatus

Seven, right-handed males served as participants [age 23 ± 2 (SD) years, height 180 ± 5 (SD) cm, weight 70 ± 3 (SD) kg]. None reported any musculoskeletal or neurological disorders and all refrained from alcohol for 24 hours before the experiment. All participants gave written informed consent and the study was approved by the research ethics committee in College of Life and Environmental Sciences at the University of Birmingham. All experiments were performed in accordance with their guidelines and regulations.

The participants stood barefoot on the cantilevered springboard that was limited to compliant motion in the vertical direction (Fig. 1) and was positioned 90 cm in front of a back-projected visual display. They stood with their feet spaced hip-width apart, at a self selected foot angle. The effective stiffness of the springboard was 4.08 N/mm (when loaded centrally), which is within the range of branch stiffnesses found in tropical forest trees^59^. Complaint branches can deflect in all directions, particularly in windy weather. However when loaded by the weight of a large bodied ape, by far the greatest deflections will be in the vertical direction. The participants stood facing the fulcrum of the springboard so they could not see when we applied a mechanical perturbation to the free end of the springboard at a random time in the experiment to destabilise the body.

Body sway was recorded by a 12 camera optoelectronic motion capture system (Oqus, Qualisys, Sweden) which tracked the position of a reflective marker attached to the skin overlying the 7th cervical vertebrae (C7). Electromyographic (EMG) data was recorded for the soleus (SOL), vastus lateralis (VL) and rectus femoris (RF) muscles in the right leg. Data was collected via Ag–AgCl surface electrodes with a 10 mm diameter conductive area and an inter-electrode distance of 20 mm (Dual Electrode, Noraxon, Scottsdale, AZ, USA, after^60^. EMG signals were transmitted wirelessly to an amplifier system (ZeroWire, Aurion, Italy), amplified (×1000), digitized, sampled at 1 kHz and stored together with the kinematic and CoP data for off-line analysis. All data streams were synchronised using a single common trigger and recorded using a single analogue to digital converter. The EMG system recorded at 1000 Hz and the camera system recorded at 200 Hz.

Once the participants were standing quietly the mechanical perturbation was introduced via a computer controlled linear actuator system (XTR 2504, Copley Controls Corp, USA) connected to the free end of the spring board via a single axis force transducer (F250, Novatech, UK). The actuator was controlled via custom software written within Labview 2009 (National Instruments, Newbury, UK) which used feedback from the sensor in order to track the movement of the board in a zero force mode. Once triggered the actuator applied a vertical load of 100 ± 3 (SD) N displacing the board vertically down, thereby generating a substantial downwards and small backwards perturbation to the participants. The actuator’s TTL trigger signal was simultaneously recorded with the force and EMG data via the analogue to digital converter within the motion tracking system allowing the onset of the perturbation to be determined.

During the experiments participants wore goggles that limited their visual field to 76 ± 10 (SD)° in the horizontal plane and 72 ± 10 (SD)° in the vertical plane centred on their direction of forward gaze; this ensured that the visual display screen encompassed their foveal field of view and also part of the forward projecting extra-foveal field. The video provided a two dimensional representations of branch movement that eliminated the use of possible stereoscopic cues. We applied both these constraints to ensure that the most salient visual cues affecting balance were present in the participants’ fields of view. Thus, although the impact of visual stimuli differs depending on whether environmental motion is detected within the central or the peripheral visual field, it is forward (foveal) vision that has the highest acuity and that underlies the detection of rapid object movements^61^. Also, the detection of the direction of movement of an object, and the time to contact, are provided by information extracted directly from the optical flow field without the necessity of stereoscopic cues^62^. Radial optic flow as caused by a looming object or during forward locomotion, however, has an effect on balance only when presented in the centre of the visual field^63, 64^. In contrast, laminar optic flow (parallel flow lines) has been shown to have impact on balance irrespective of the region in the visual field, both in the centre and the periphery^63, 64^. Laminar optic flow is much more prominent in the video for the DVE condition due to left-right, up-down and diagonal movements of the tree branches. For example, a prominent leafy branch in the centre foreground of the image moved with a lateral amplitude of 463 ± 38 (SD) mm at an inclination angle of 34 ± 8 (SD)° to the horizontal. Overall, the branches shown in the video moved in a multi directional pattern with semi regular frequency of 0.4 ± 0.05 (SD) Hz. Thus, the DVE condition induced considerable environmental noise in the visual channel and therefore should have had a pronounced effect on body sway.

To study the role of light touch a 21 mm diameter carbon fibre hand support was mounted at the shoulder (acromion) height of each participant on a 6 degree of freedom force sensor (Delta, ATI, Apex, NC, USA) in a cantilever arrangement, and positioned 310 mm to the side of the centre line of the spring board in parallel with the participant, to replicate an adjacent branch at approximately shoulder level height (Fig. 1). The attenuating effects of light touch are greater when the finger is positioned in the plane of greatest sway^27^. The participants therefore made contact with the hand support by placing the index finger of their right hand on a marker situated 450 mm in front of their body, which was found to be a comfortable location for all participants. When loaded at this point the pole had an effective stiffness of 1.17 N/mm. The contact point was not visible to the participant due to the visual field restrictions.

For the light touch experiments, participants were asked to maintain a “light touch with the hand support with just enough force to maintain contact”. During tests with no touch conditions the participants were asked to maintain a similar posture to the touch conditions but to move their hand slightly to the side in order to avoid making contact with the pole. All possible combinations of the three variables (vision, touch and compliance) were tested resulting in 12 conditions, with 10 trials of each condition. Trials were presented in two counterbalanced blocks. Before testing, participants were encouraged to practise standing on the board as it was perturbed, although none asked to experience more than 4 perturbations. Participants were instructed to stand relaxed on the springboard without moving and were asked to say when they had reached a stable postural state, at which point the data recording for each trial would start. Each trial lasted 20 seconds. For trials on the compliant surface the participants were subjected to a single perturbation at a randomized time interval between 5 and 14 seconds after the start of the recording to ensure they were unaware of the exact timing of the perturbation.

### Data Analysis

Individual data streams were analyzed using custom interactive software written in MatLab R2008a (Mathworks, Inc., Natick, MA, USA). The antero-posterior (AP) component of the kinematics of a marker placed on the 7^th^ cervicular vertebrae was digitally low-pass filtered at 10 Hz (dual pass 4th-order Butterworth filter) and differentiated to obtain rate-based measures of change per second (dC7).

To investigate the time course of the balance response following the springboard perturbation, each trial was segmented into bins of 1 s duration from 3 s before to 6 s after the onset of the perturbation. The within-bin standard deviation (SD) of the rate of change parameters was determined for each time bin in the AP direction^65^. The time course data for each trial was then divided into two phases: the baseline phase before the perturbation (t < 0 s) and the stabilisation phase after the perturbation (t > 0 s).

The onsets of the perturbations were determined using the actuators TTL trigger signal linked to the EMG. EMG recordings were band-pass filtered between 10 and 500 Hz, rectified and smoothed by a moving average with 15 ms width to obtain the EMG envelope. The EMG envelopes were normalized against the respective muscle activities during maximum voluntary contraction (MVC) of each specific muscle obtained for each participant^60^. Mean EMG values were extracted for the base-line phase before the perturbation^8^. For the post-perturbation stabilization phase muscle contraction onsets were defined as the time point at which the rectified EMG amplitude increased by 4 standard deviations above a mean baseline period^65^.

The data for the base line and stabilization phases was subjected to repeated-measures analysis of variance (ANOVA), performed in SPSS 16. The significance threshold was set to P = 0.05 after Greenhouse-Geisser correction. Vision, compliance and touch conditions were primary independent factors and time course was an additional factor for the post perturbation analysis. Significant interactions were explored with post hoc single comparison.
